# Publisher Correction: Amazonian understory forests change phosphorus acquisition strategies under elevated CO_2_

**DOI:** 10.1038/s41467-026-72992-7

**Published:** 2026-05-08

**Authors:** Nathielly P. Martins, Lucia Fuchslueger, Laynara F. Lugli, Oscar J. Valverde-Barrantes, Richard J. Norby, Iain P. Hartley, Izabela Aleixo, Fabricio B. Baccaro, Barbara N. S. Brum, Crisvaldo Cássio Silva de Souza, Carine M. Cola, Raffaello Di Ponzio, Amanda Damasceno, Tomas F. Domingues, Vanessa R. Ferrer, Katrin Fleischer, Sabrina Garcia, Alacimar Guedes, Florian Hofhansl, David M. Lapola, Juliane G. Menezes, Anna C. M. Moraes, Ana Caroline Miron, Leonardo Ramos de Oliveira, Cilene Palheta, Iokanam S. Pereira, Maria Pires Martins, Gyovanni Ribeiro, Jéssica Schmeisk-Rosa, Anja Rammig, Flavia D. Santana, Yago R. Santos, Lara Siebert Silva, Bruno Takeshi T. Portela, Gabriela Ushida, Carlos A. Quesada

**Affiliations:** 1 Professorship of Land Surface–Atmosphere Interactions, TUM School of Life Sciences, Technical University of Munich, Freising, Germany; 2 Coordination of Environmental Dynamics, National Institute for Amazonian Research, Manaus, Brazil; 3 Center for Microbiology and Environmental Systems Science, University of Vienna, Vienna, Austria; 4 Department of Biological Sciences, Institute of Environment, International Center of Tropical Botany, Florida International University, Miami, FL USA; 5 School of Geography, Earth & Environmental Sciences, University of Birmingham, Edgbaston, UK; 6 Environmental Sciences Division, Oak Ridge National Laboratory, Oak Ridge, TN USA; 7 Geography, Faculty of Environment, Science and Economy, University of Exeter, Exeter, UK; 8 Coordination of Technology and Innovation, National Institute for Amazonian Research, Manaus, Brazil; 9 Department of Biology, Federal University of Amazonas, Manaus, Brazil; 10 Programa de Pos-graduação em Ecologia, Conservação e Manejo da Vida Silvestre, Instituto de Ciências Biologicas, Universidade Federal de Minas Gerais, Belo Horizonte, Brazil; 11 Departamento de Biologia, Faculdade de Filosofia, Ciências e Letras de Ribeirão Preto, University of São Paulo, Ribeirão Preto, Brazil; 12 Systems Ecology, Amsterdam Institute for Life and Environment, Vrije Universiteit Amsterdam, Amsterdam, The Netherlands; 13 International Institute for Applied Systems Analysis, Laxenburg, Austria; 14 Universidade Estadual de Campinas – UNICAMP, Campinas, Brazil; 15 Department of Biology, University of Hamburg, Hamburg, Germany; 16 Chico Mendes Institute for Biodiversity Conservation (ICMBio), Brasília, Brazil; 17 Forest Ecology and Forest Management, Wageningen University & Research, Wageningen, The Netherlands

**Keywords:** Climate-change ecology, Tropical ecology, Element cycles

Correction to: *Nature Communications* 10.1038/s41467-026-72098-0, published online 28 April 2026

In this article, the captions to Figs. 1–4 were inadvertently truncated. The original article has been corrected.

