# Supplementary material for: Amazonian understory forests change phosphorus acquisition strategies under elevated CO2
Source: Nat Commun. 2026 Apr 28;17:3740. doi: 10.1038/s41467-026-72098-0 (PMC13125542; doi:10.1038/s41467-026-72098-0)
Supplement: Supplementary file 1 — Supplementary information [file 41467_2026_72098_MOESM1_ESM.pdf]

Supplementary information

Amazonian understory forest change phosphorus acquisition strategies under elevated CO_2

Nathielly P. Martins*, Lucia Fuchslueger*, Laynara F. Lugli, Oscar J. Valverde-Barrantes, Richard J. Norby, Iain P. Hartley, Izabela Aleixo, Fabricio B. Baccaro, Barbara N.S. Brum, Crisvaldo Cássio Silva de Souza, Carine M. Cola, Raffaello Di Ponzio, Amanda Damasceno, Tomas F. Domingues, Vanessa R. Ferrer, Katrin Fleischer, Sabrina Garcia, Alacimar Guedes, Florian Hofhansl, David M. Lapola, Juliane G. Menezes, Anna C. M. Moraes, Ana Caroline Miron, Leonardo Ramos de Oliveira, Cilene Palheta, Iokanam S. Pereira, Maria Pires, Gyovanni Ribeiro, Jéssica Schmeisk-Rosa, Anja Rammig, Flavia D. Santana, Yago R. Santos, Lara Siebert Silva, Bruno Takeshi T. Portela, Gabriela Ushida and Carlos A. Quesada.

***Authors for correspondence:**

Nathielly P. Martins: nathielly.martins@tum.de

Lucia Fuchslueger: lucia.fuchslueger@univie.ac.at

Table S1 | Open-top chambers experiment (OTC), soil and plant description. The baseline description was conducted for each OTC, providing the mean and standard error (n=4) for the respective treatments of ambient CO_2 (aCO_2) and elevated CO_2 (eCO_2). For the soil data, the values are the average of two soil cores collected per OTC, and for plant height and diameter, they are the average of total plant individuals present inside each OTC. The baseline collections were conducted on different dates in 2019 (see Figure S2 for details).

	aCO_2					eCO_2				
	P1	P2	P3	P4		P1	P2	P4	P3	
	1	8	9	X	Mean ± SE	2	4	5	Y	Mean ± SE
pH		3.73	3.81	3.66	3.74 ± 0.06		3.71	3.76	3.82	3.76 ± 0.04
Clay (%)		65.31	62.68	61.47	63.15 ± 1.70		56.95	62.75	62.27	61.99 ± 2.31
Sand (%)		12.57	12.03	12.31	12.30 ± 0.34		12.95	11.77	12.74	12.49 ± 0.55
Silt (%)		22.11	25.28	26.21	24.53 ± 1.83		30.10	25.47	20.97	25.51 ± 2.26
Carbon (%)	3.69	3.03	3.23	2.70	3.16 ± 0.18	2.50	2.64	3.08	2.95	2.79 ± 0.17
Nitrogen (%)	0.28	0.25	0.26	0.21	0.25 ± 0.01	0.20	0.22	0.23	0.23	0.22 ± 0.01
Total P	82.93	103.05	104.70	127.02	104.43 ± 8.12	79.90	83.49	93.68	129.50	96.64 ± 10.68
Organic P	25.65	24.19	29.10	27.37	26.58 ± 1.71	23.85	22.81	25.03	29.20	25.22 ± 1.40
Inorganic P	21.99	34.94	28.55	24.73	27.55 ± 3.09	21.75	21.20	24.50	26.69	23.58 ± 1.34
Residual P	35.29	43.92	47.04	74.91	50.29 ± 6.90	34.29	39.47	44.15	73.60	47.88 ± 9.28
Plant individuals	18	14	18	8	14.50 ± 2.21	13	13	13	10	12.25 ± 0.75
Species richness	9	11	13	6	9.75 ± 1.50	12	7	11	4	8.50 ± 1.85
Height (cm)	93.86	86.18		158.7	112.91 ± 23.27	108.5	112.6	132.3	110	115.85 ± 5.60
Diameter (mm)	11.76	10.01	11.1	13.58	11.61 ± 0.74	13.0	11.1	13.5	10.5	12.03 ± 0.72

The total phosphorus (P) and the other fractions of P (organic, inorganic, and residual) are shown by mg kg^{-1}. The data for plant individuals, height, and diameter refer to all individuals within OTCs (including those not identified). The pairs description (i.e., P1, P2, P3, P4) refers to the OTC pairs used to control the increase in CO_2 using an ambient CO_2 OTC as a reference; the respective OTC pairs were also used as random factors in the statistical analyses. For more details about the plan's species composition, refer to Damasceno et al. (2024).

Table S2 | Statistical analysis of elevated CO_2 effects on fine root nutrient acquisition mechanisms in the litter layer and soil. Data were analyzed using generalized linear mixed models, with a specific model for each sampling date and variable. The treatment effect was included as a fixed factor, while spatial variability was accounted for by incorporating OTC pairs as a random factor. Baseline collections were conducted in August 2019, before CO_2 enrichment, to characterize natural spatial variability. The "elevated CO_2 experiment" corresponds to eight months of CO_2 enrichment for roots in the litter layer and 12 months for roots in the soil (see Fig. S2 for details). The delta CO_2 effect was calculated as the difference between post-enrichment collections (i.e., after eight months in the litter layer and 12 months in the soil) and baseline measurements for both treatments (i.e., ambient CO_2 treatment (aCO_2) - ambient CO_2 baseline, and eCO_2 treatment – eCO_2 baseline; see methods section).

	Baseline						Elevated CO_2 experiment						Delta CO_2 effect					
Variable	Est.	Std. error	*z*	*P*	R^2m	R^2c	Est.	Std. error	*z*	*P*	R^2m	R^2c	Est.	Std. error	*z*	*P*	R^2m	R^2c
**Litter layer**																		
Productivity (mg cm^{-2} yr^{-1})	0.31	01.7	1.76	0.07	0.07	0.84	-0.01	1.02	-0.01	0.98	0.00	0.11	-0.33	0.91	-0.36	0.71	0.01	0.13
SRL (m g^{-1})	**-9.88**	**4.84**	**-2.04**	**0.04**	**0.40**	**0.40**	31.56	17.87	1.76	0.07	0.34	0.34	**45.0**	**20.30**	**2.22**	**0.02**	**0.49**	**0.49**
SRA (cm^2 g$^{-1)}$	-0.23	0.18	-1.30	0.19	0.21	0.21	**0.44**	**0.21**	**2.04**	**0.04**	**0.40**	**0.40**	273.0	161.2	1.69	0.09	0.36	0.36
RTD (g cm^{-3})	-0.03	0.03	-0.82	0.41	0.10	0.10	0.03	0.04	-0.72	0.46	0.08	0.08	0.01	0.08	0.15	0.87	0.00	0.00
Diameter (mm)	**0.06**	**0.02**	**2.35**	**0.01**	**0.22**	**0.78**	**-0.09**	**0.03**	**-2.57**	**0.009**	**0.52**	**0.53**	**-0.16**	**0.03**	**-4.64**	**<0.001**	**0.81**	**0.81**
APase (nmol mg^{-1} h^{-1})							0.37	0.54	0.68	0.49	0.07	0.07						
APase (nmol cm^{-1} h^{-1})							0.07	0.18	0.40	0.68	0.02	0.02						
APase (nmol cm^{-2} h^{-1})							0.16	0.20	0.80	0.42	0.09	0.09						
**Soil depth**																		
Biomass (mg cm^2)	**-0.53**	**0.22**	**-2.43**	**0.01**	**0.06**	**0.91**	-0.06	0.48	-0.14	0.88	0.00	0.00	0.47	0.35	1.34	0.18	0.03	0.86
Productivity (mg cm^{-2} yr^{-1})	-0.83	1.54	-0.54	0.58	0.04	0.04	**-0.16**	**0.26**	**-4.45**	**<0.001**	**0.47**	**0.84**	**-13.31**	**6.95**	**-1.91**	**0.05**	**0.34**	**0.34**
SRL (m g^{-1})	**1.10**	**0.32**	**3.38**	**<0.001**	**0.56**	**0.70**	**10.64**	**4.32**	**2.46**	**0.01**	**0.04**	**0.94**	*-9.87	23.83	-0.41	0.67	0.02	0.97
SRA (cm^2 g^{-1})	**0.77**	**0.22**	**3.46**	**<0.001**	**0.62**	**0.65**	0.09	0.09	1.02	0.31	0.02	0.84	*-168.5	193.5	-0.87	0.38	0.09	0.99
RTD (g cm^{-3})	**-0.07**	**0.03**	**-1.96**	**0.04**	**0.35**	**0.35**	0.00	0.02	0.16	0.87	0.00	0.11	**0.07**	**0.03**	**2.40**	**0.01**	**0.41**	**0.41**
Diameter (mm)	**-0.17**	**0.06**	**-2.92**	**0.003**	**0.33**	**0.72**	-0.15	0.11	-1.37	0.17	0.18	0.30	0.02	0.08	0.30	0.75	0.00	0.75
APase (nmol mg^{-1} h^{-1})	6.97	4.63	1.50	0.13	0.24	0.24	44.44	39.89	1.11	0.26	0.15	0.15	0.51	0.38	1.35	0.17	0.19	0.26
APase (nmol cm^{-1} h^{-1})	1.91	1.15	1.65	0.09	0.28	0.28	2.54	2.78	0.91	0.36	0.03	0.70	0.63	3.72	0.16	0.86	0.00	0.44
APase (nmol cm^{-2} h^{-1})	7.59	6.51	1.16	0.24	0.16	0.16	**36.73**	**13.04**	**2.81**	**0.004**	**0.10**	**0.90**	29.13	18.57	1.56	0.11	0.07	0.78
*Mycorrhiza colonization (%)													**30.25**	**9.65**	**3.13**	**0.001**	**0.43**	**0.43**

The models are fitted using maximum likelihood estimation, and statistically significant results are reported at the 95% confidence level and shown in boldface (significant effects, $P < 0.05$). We use the glmmTMB package to run the statistical models, and the model parameters are presented for each variable. Where: Est. = model estimate; Std. error = standard error; Z = wald statistic; P = p-value; R^2m = variance explained by fixed effects; R^2c = variance explained by fixed + random effects. *Models use the OTC identity as a random factor to obtain better adjustment. SRL, specific root length; SRA, specific root area; RTD, root tissue density; APasse, acid phosphomonoesterase activity (phosphatase). For root phosphatase variables in the litter layer and mycorrhiza colonization in the soil, baseline sampling was not possible. Thus, we analyze the effect of eCO_2 by comparing the aCO_2 levels after 8 and 12 months under eCO_2 for the litter and soil variables, respectively.

Table S3 | Statistical analysis of elevated CO_2 effects on soil nutrient concentrations. Data were analyzed using generalized linear mixed models, with a specific model for each sampling date and variable. The treatment effect was included as a fixed factor, while spatial variability was accounted for by incorporating open-top chamber OTC pairs as a random factor. Baseline collections were conducted in February 2019 for P variables and in August 2019 for other elements before CO_2 enrichment to characterize natural spatial variability. The "elevated CO_2 experiment" corresponds to measurements taken after 12 months of CO_2 enrichment. The delta CO_2 effect was calculated as the difference between post-enrichment collections and baseline measurements for both treatments (i.e., ambient CO_2 treatment (aCO_2) - ambient CO_2 baseline, and eCO_2 treatment – eCO_2 baseline; see methods section).

		Baseline						Elevated CO_2 experiment						Delta CO_2 effect					
		Est.	Std. error	*z*	*P*	R^2m	R^2c	Est.	Std. error	*z*	*P*	R^2m	R^2c	Est.	Std. error	*z*	*P*	R^2m	R^2c
Carbon (%)		-0.37	0.21	-1.72	0.08	0.29	0.29	-0.30	0.28	-1.08	0.28	0.14	0.14	0.10	0.27	0.38	0.70	0.020	0.024
Nitrogen (%)		-0.02	0.01	-1.81	0.06	0.31	0.31	-0.01	0.01	**-0.79**	0.42	0.07	0.18	0.01	0.01	0.68	0.49	0.05	0.23
Total P		-7.78	10.82	-0.71	0.47	0.05	0.29	**-7.35**	**2.26**	**-3.25**	**0.001**	**0.45**	**0.69**	*0.43	11.19	0.03	0.96	0.00	0.51
P_t	$NaHCO_3$	-0.76	0.48	-1.59	0.11	0.12	0.65	-0.84	1.35	-0.62	0.53	0.02	0.50	-0.07	1.71	-0.04	0.96	0.00	0.47
	NaOH	**-3.53**	**1.43**	**-2.46**	**0.01**	**0.32**	**0.63**	**-12.24**	**2.59**	**-4.72**	**<0.001**	**0.53**	**0.83**	***-8.70**	**3.49**	**-2.49**	**0.01**	**0.47**	**0.73**
P_i	Resin	-0.93	0.72	-1.28	0.2	0.19	0.19	**-0.69**	**0.21**	**-3.25**	**0.001**	**0.60**	**0.60**	0.23	0.72	0.32	0.74	0.014	0.014
	$NaHCO_3$	**-0.68**	**0.29**	**-2.30**	**0.02**	**0.35**	**0.53**	-0.84	0.49	-1.69	0.09	0.27	0.33	-0.16	0.63	-0.25	0.79	0.00	0.12
	NaOH	-2.26	1.63	-1.38	0.16	0.21	0.21	**-3.25**	**0.94**	**-3.44**	**<0.001**	**0.50**	**0.70**	-0.98	0.97	-1.01	0.31	0.12	0.12
	HCl	-0.13	0.22	-0.60	0.54	0.04	0.06	**0.18**	**0.02**	**6.26**	**<0.001**	**0.24**	**0.95**	0.31	0.20	1.55	0.11	0.25	0.25
P_o	NaHCO3	-0.08	0.42	-0.19	0.84	0.00	0.52	0.43	1.17	0.37	0.70	0.01	0.36	0.46	1.61	0.28	0.77	0.00	0.29
	NaOH	**-1.27**	**0.41**	**-3.06**	**0.002**	**0.14**	**0.89**	-8.98	2.68	**-3.34**	**<0.001**	**0.45**	**0.71**	***-7.71**	**2.63**	**-2.93**	**0.003**	**0.55**	**0.77**
Total organic P		**-1.35**	**0.45**	**-2.99**	**0.002**	**0.10**	**0.92**	**-9.071**	**3.20**	**-2.83**	**0.004**	**0.53**	**0.53**	**-7.72**	**2.59**	**-2.97**	**0.002**	**0.55**	**0.55**
Total inorganic P		-4.01	2.66	-1.50	0.13	0.24	0.24	**-4.61**	**1.36**	**-3.38**	**<0.001**	**0.57**	**0.64**	-0.59	1.90	-0.31	0.75	0.01	0.01
Extractable P		**-5.37**	**3.23**	**-1.90**	**0.05**	**0.28**	**0.45**	-13.82	2.87	**-4.80**	**<0.001**	**0.70**	**0.78**	**-8.33**	**4.37**	**-1.90**	**0.05**	**0.34**	**0.34**
Residual P		-2.41	10.15	-0.23	0.81	0.00	0.09	**6.21**	**1.49**	**4.16**	**<0.001**	**0.30**	**0.87**	*7.31	12.38	0.59	0.55	0.04	0.52

The models are fitted using maximum likelihood estimation, and statistically significant results are reported at the 95% confidence level and shown in boldface (significant effects, $P < 0.05$). We use the glmmTMB package to run the statistical models, and the model parameters are presented for each variable. Where: Est. = model estimate; Std. error = standard error; Z = wald statistic; P = p-value; R^2m = variance explained by fixed effects; R^2c = variance explained by fixed + random effects. *Models use the OTC identity as a random factor to obtain better adjustment. The total phosphorus (P) and the P fractions are shown in mg kg^{-1}. The resin and HCl fractions are obtained first using an anion exchange membrane and hydrogen chloride. The $NaHCO_3$ represents the total (Pt), inorganic (Pi), and Organic (Po) P fractions resulting from bicarbonate extraction, and the NaOH represents the total (Pt), inorganic (Pi), and Organic (Po) P fractions resulting from hydroxide extraction. Total organic and inorganic P represents the sum of their respective fractions of $NaHCO_3$ and NaOH. The extractable P represents the total P, which may be represented by the sum of the organic and inorganic fractions ($NaHCO_3$ and NaOH) plus the resin and HCl, and the residual P is the difference between the total and the extractable P.

Table S4 | Statistical analysis of elevated CO_2 effects on soil microbial activity. Data were analyzed using generalized linear mixed models, with a specific model for each sampling date and variable. The treatment effect was included as a fixed factor, while spatial variability was accounted for by incorporating open-top chamber OTC pairs as a random factor. Baseline collections were conducted in August 2019, before CO_2 enrichment, to characterize natural spatial variability. The "elevated CO_2 experiment" corresponds to measurements taken after 12 months of CO_2 enrichment (November 2020). The delta CO_2 effect was calculated as the difference between post-enrichment collections and baseline measurements for both treatments (i.e., ambient CO_2 treatment (aCO_2) - ambient CO_2 baseline, and eCO_2 treatment – eCO_2 baseline; see methods section).

	Baseline						Elevated CO_2 effect						Delta CO_2 effect					
	Est.	Std. error	Z	*P*	R^2m	R^2c	Est.	Std. error	Z	*P*	R^2m	R^2c	Est.	Std. error	Z	*P*	R^2m	R^2c
Microbial C	*43.40	119.23	0.36	0.71	0.01	0.01	**-77.8**	**22.32**	**-3.48**	**<0.001**	**0.35**	**0.79**	*-99.44	135.8	-0.73	0.46	0.07	0.08
Microbial N	-1.50	1.49	-1.00	0.31	0.03	0.75	-4.74	3.29	-1.44	0.15	0.08	0.71	-1.83	5.04	-0.36	0.71	0.18	0.18
Microbial P	**-1.02**	**0.49**	**-2.06**	**0.03**	**0.37**	**0.37**	**-0.65**	**0.30**	**-2.11**	**0.03**	**0.28**	**0.55**	0.48	0.50	0.95	0.33	0.11	0.11
Microbial C: N ratio	0.70	2.34	0.30	0.76	0.01	0.17	0.03	1.96	0.01	0.98	0.00	0.00	-0.66	1.45	-0.45	0.64	0.01	0.60
Microbial C:P ratio	48.0	57.25	0.83	0.40	0.05	0.40	16.18	38.23	0.42	0.67	0.01	0.50	-47.97	40.41	-1.18	0.23	0.03	0.83
Microbial N: P ratio	11.73	11.18	1.05	0.29	0.13	0.13	8.12	6.62	1.22	0.21	0.09	0.55	-13.68	9.71	-1.40	0.15	0.22	0.22
CB	**5.69**	**1.75**	**3.24**	**0.001**	**0.29**	**0.80**	-0.79	0.77	-1.02	0.30	0.09	0.37	**-6.64**	**1.07**	**-6.17**	**<0.001**	**0.49**	**0.90**
BG	**2.80**	**0.87**	**3.20**	**0.001**	**0.59**	**0.59**	**-0.63**	**0.21**	**-2.89**	**0.003**	**0.54**	**0.54**	**-3.43**	**0.88**	**-3.87**	**<0.001**	**0.68**	**0.68**
NAG	1.85	3.33	0.55	0.57	0.04	0.04	0.03	0.24	0.14	0.88	0.00	0.91	-1.82	3.8	-0.48	0.63	0.03	0.03
AP	-0.09	0.33	-0.30	0.76	0.01	0.01	-0.21	0.12	-1.73	0.08	0.29	0.29	0.20	118.8	0.00	0.99	0.00	0.00
C: NAG ratio (log)	0.11	0.22	0.54	0.58	0.04	0.04	-0.01	0.43	-0.03	0.96	0.00	0.07	0.01	0.40	0.04	0.96	0.00	0.57
C: AP ratio (log)	**0.14**	**0.04**	**3.11**	**0.001**	0.44	0.67	-0.03	0.02	-1.57	0.11	0.19	0.45	**-0.18**	**0.03**	**-4.89**	**<0.001**	**0.71**	**0.79**
N: P ratio (log)	**0.09**	**0.04**	**1.83**	**0.06**	0.24	0.48	0.01	0.01	1.57	0.11	0.02	0.92	-0.07	0.05	-1.37	0.16	0.10	0.61
C enzyme: Mic. C ratio	0.03	0.03	1.16	0.24	0.14	0.25	-0.00	0.00	-0.20	0.83	0.00	0.50	-0.04	0.03	-1.28	0.19	0.17	0.26
N enzyme: Mic. C ratio	0.01	0.008	1.27	0.20	0.05	0.75	**0.002**	**0.00**	**2.18**	**0.02**	**0.19**	**0.71**	-0.008	0.008	-0.97	0.33	0.03	0.73
P enzyme: Mic: C ratio	-0.35	0.31	-1.11	0.26	0.10	0.41	0.02	0.03	0.80	0.42	0.05	0.37	0.36	0.30	1.22	0.21	0.10	0.49

The models are fitted using maximum likelihood estimation, and statistically significant results are reported at the 95% confidence level and shown in boldface (significant effects, $P < 0.05$). We use the glmmTMB package to run the statistical models, and the model parameters are presented for each variable. Where: Est. = model estimate; Std. error = standard error; Z = wald statistic; P = p-value; R^2m = variance explained by fixed effects; R^2c = variance explained by fixed + random effects. *Models use the OTC identity as a random factor to obtain better adjustment. Total of carbon (C), nitrogen (N), and phosphorus (P) immobilized on microbial biomass and its stoichiometric ratio. The cellobiosidase (CB) and β-1,4-glucosidase (BG) are enzymes responsible for hydrolyzing the carbon, β-1,4-N-acetylglucosaminidase (NAG) for the nitrogen, and

acid phosphatase (AP) for the organic phosphorus. Enzyme stoichiometry was calculated using the log ratio of activity rates. The carbon enzymes are represented by the sum of cellobiosidase (CB) and β-1,4-glucosidase (BG) activity. The ratio of CNP enzymes and CNP microbial biomass obtained the stoichiometry of enzymes and microbial biomass.

.

Table S5- Elevated CO_2 effect on leaf litter decomposition and nutrient dynamics after one year of the field experiment. Statistical parameters are the results of Generalized linear mixed models for each variable. The model considers the effect of the treatment as a fixed factor and controls the spatial variability using the OTC's pairs as a random factor.

	Fixed factor coefficients					
	Estimate	Std. error	*z*	*P*	R^2m	R^2c
Litter mass loss (%)	-2.07	5.86	-0.35	0.72	0.01	0.01
Carbon (%)	-2.46	2.13	-1.15	0.24	0.09	0.48
Nitrogen (%)	-0.16	0.09	-1.77	0.07	0.23	0.49
Phosphorus g kg^{-1}	**-0.03**	**0.01**	**-2.50**	**0.01**	**0.29**	**0.67**

The models are fitted using maximum likelihood estimation, and statistically significant results are reported at the 95% confidence level and shown in boldface (significant effects, $P < 0.05$). We use the glmmTMB package to run the statistical models, and the model parameters are presented for each variable. Where: Est. = model estimate; Std. error = standard error; Z = wald statistic; P = p-value; R^2m = variance explained by fixed effects; R^2c = variance explained by fixed + random effects.

Table S6 | Average of key root parameters and litter and soil nutrient concentration. Data is presented by collection time, where: Baseline collections were conducted before CO_2 enrichment, to characterize natural spatial variability. The "elevated CO_2 experiment" corresponds to measurements taken after the start of CO_2 enrichment. The delta CO_2 effect was calculated as the difference between post-enrichment collections and baseline measurements for both treatments (i.e., ambient CO_2 treatment (aCO_2) - ambient CO_2 baseline, and eCO_2 treatment – eCO_2 baseline; see methods section). The root parameters represent C investment in root productivity, foraging strategies (i.e., SRL and AMF) and mining strategy represented by the root APase potential activity. In addition, the leaf and soil nutrient carbon (C), nitrogen (N) and phosphorus (P) are presented together with some of the main P fractions and P microbial activity.

			Baseline			eCO_2 experiment			Delta CO_2 response		
Layer	Variables	Unit	aCO_2	eCO_2	% Change	aCO_2	eCO_2	% Change	aCO_2	eCO_2	% Change
Litter	Root productivity	mg cm^{-2} year^{-1}	0.31	0.63	99.48	1.58	1.56	-1.05	1.26	0.93	-26.23
	SRL	m g^{-1}	24.03	15.28	-36.41	26.68	50.84	90.51	4.16	39.8	855.75
	Root APase	nmol mg^{-1} h^{-1}				111.78	126.90	13.52			
	AMF	%									
Soil	Root productivity	mg cm^{-2} year^{-1}	2.80	2.19	-21.57	19.04	4.96	-73.94	16.23	3.11	-80.84
	SRL	m g^{-1}	8.91	29.43	229.99	46.07	56.71	23.09	37.15	27.28	-26.56
	Root APase	nmol mg^{-1} h^{-1}	7.63	14.61	91.39	97.42	141.86	45.61	89.78	127.24	41.72
	AMF	%				25.75	56.0	117.47			
Litter	Total C	%				33.4	31.2	-6.58			
	Total N	%				1.66	1.50	-9.63			
	Total P	g kg^{-1}				0.38	0.34	10.52			
Soil	Total C	%	3.16	2.79	-11.72	2.98	2.73	-8.21	- 0.26	-0.06	-77.19
	Total N	%	0.25	0.22	-10.13	0.23	0.22	-3.76	-0.01	0.002	-110.92
	Total P	mg kg^{-1}	104.43	96.64	-7.45	124.21	116.85	-5.91	19.77	20.21	2.18
	Organic P	mg kg^{-1}	26.58	25.22	-5.09	39.99	26.99	-24.99	9.40	2.10	-77.61
	Inorganic P	mg kg^{-1}	27.55	23.53	-14.58	32.49	27.88	-14.19	4.93	4.34	-12.04
	Residual P	mg kg^{-1}	50.29	47.88	-4.79	57.72	61.57	10.49	5.43	8.51	56.78
	Microbial P	mg kg^{-1}	3.93	2.69	-31.53	3.24	2.59	-20.07	-0.66	0.09	-111.50

The percentage of change between elevated CO_2 (eCO_2) and ambient CO_2 (aCO_2) was calculated as: $(eCO_2 – aCO_2) /aCO_2)*100$. It is important to note that the percentage of change doesn't represent a statistical test; to see the level of significance, check the previous tables. In addition, the data presented here is from specific experiments to test a treatment effect before using the values we recommend checking the methods section.

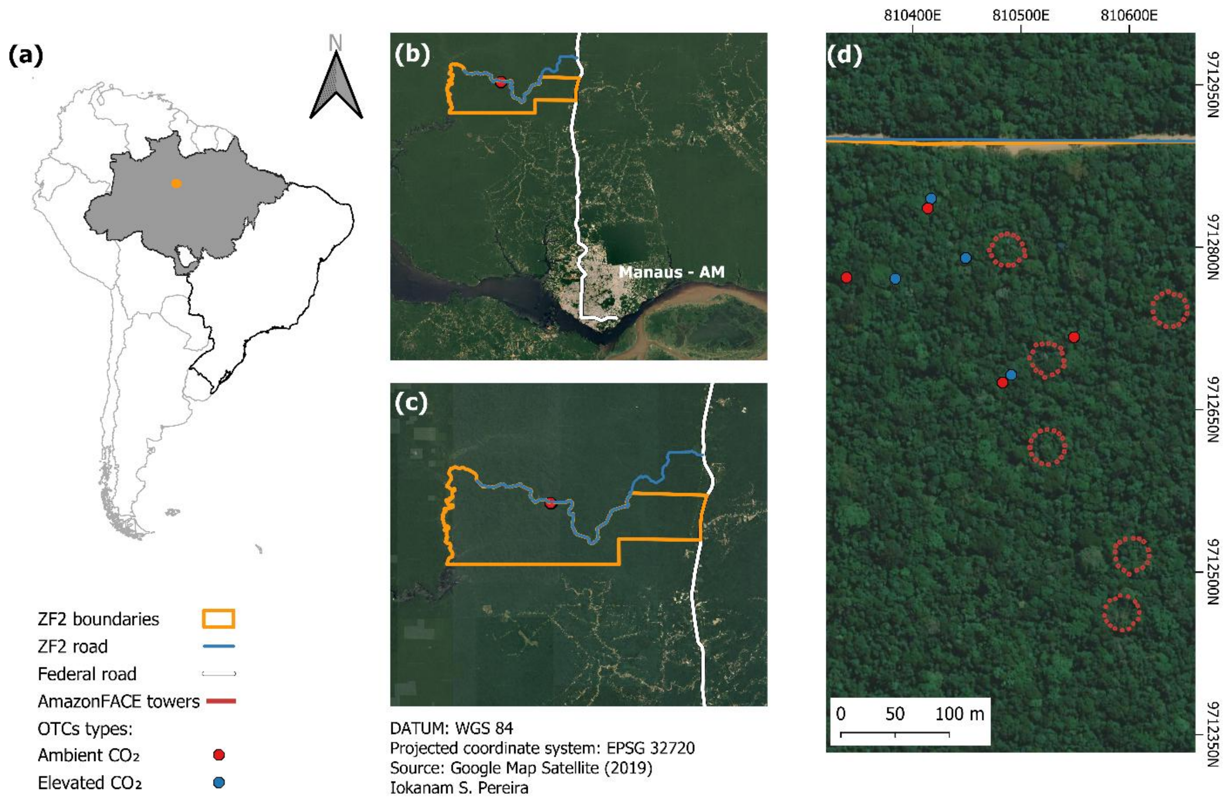

Figure S1| Spatial location of the Open Top Chambers (OTCs) experiment located at the site of the AmazonFACE program. a) Location of the ZF2 site (orange point) in central Amazonia, Brazil. (b–c) High-resolution satellite imagery showing the boundaries of the ZF2 forest reserve (orange line). Detailed view of the AmazonFACE experimental site (d), showing the distribution of OTCs: ambient CO_2 chambers (red) and elevated CO_2 chambers (blue). Dashed circles delineate the plot of the AmazonFACE program that is not being considered in this study. Adapted from Lapola et al. (2024), *AmazonFACE: 2025–2030 Science Plan* (Zenodo, DOI: 10.5281/zenodo.13770177), licensed under CC BY 4.0.

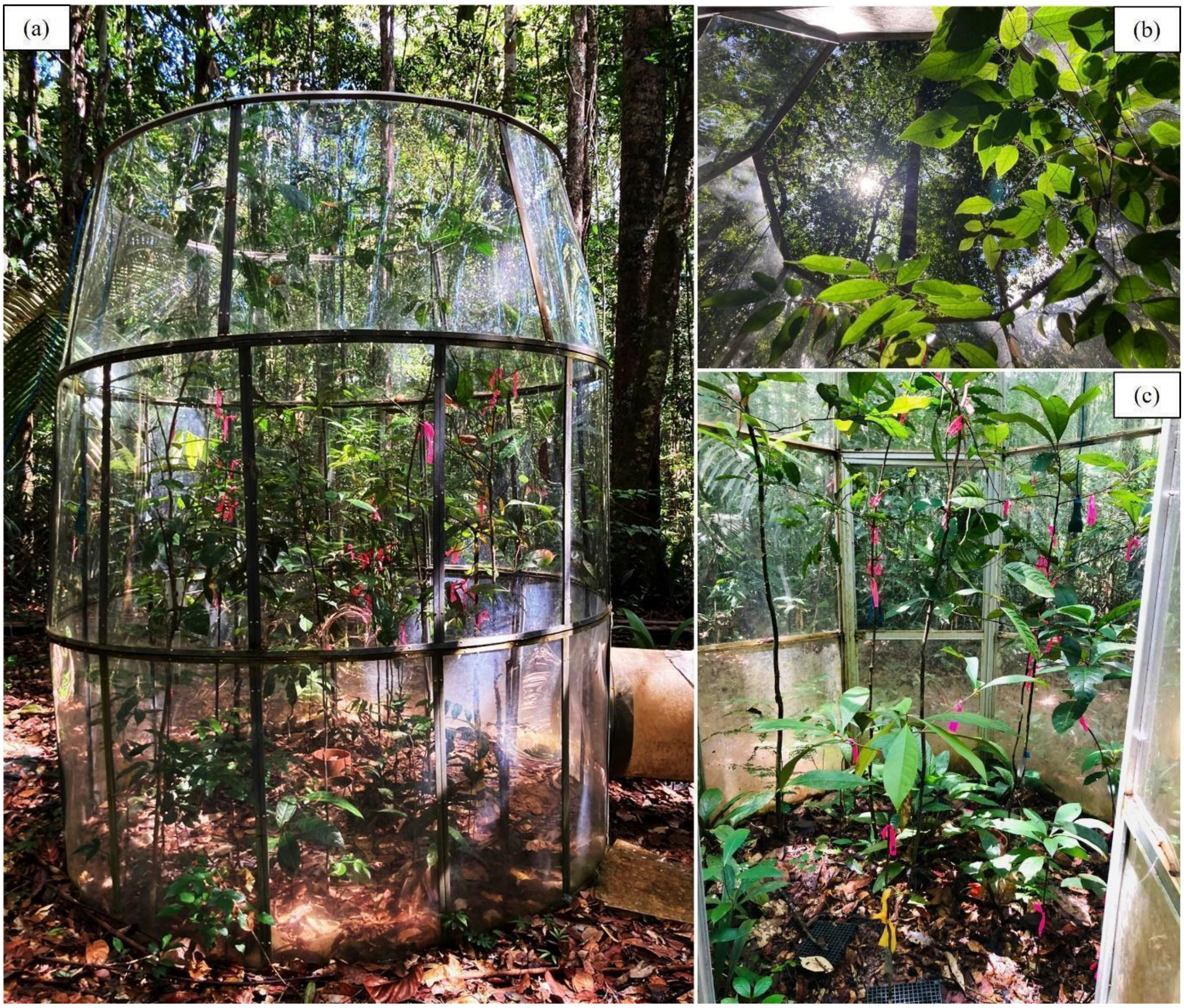

Figure S2 | Open top chamber structure. The figure illustrates (a) the design of an Open Top Chamber (OTC) structure, (b) the canopy open, and (c) the soil area used for investigating the effect of the increase in CO_2 concentrations on the belowground process. The OTC structure consists of a transparent polypropylene chamber with a 3 m height and 2.4m diameter. Images: Maria Juliana, Flavia Santana.

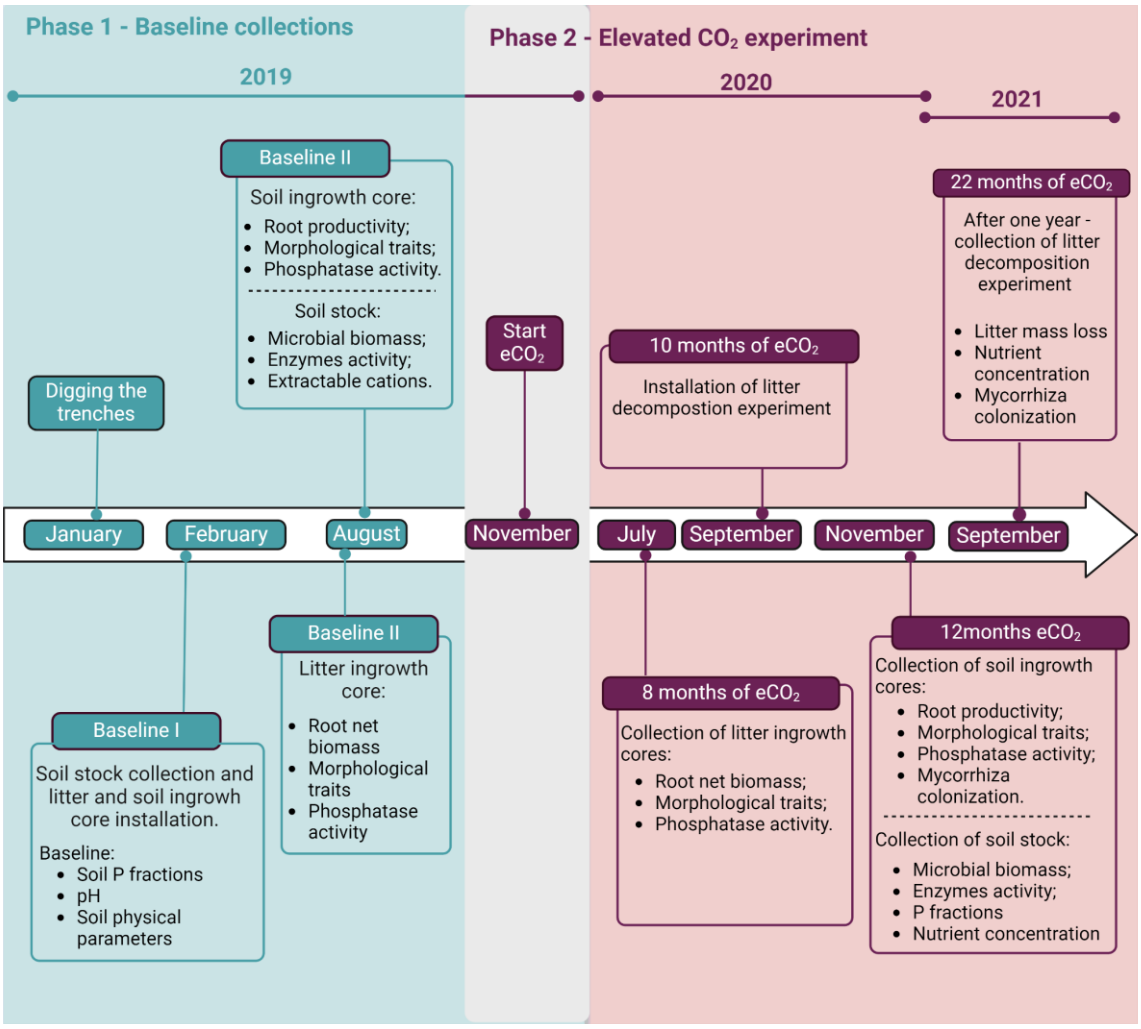

Figure S3 | Timeline of belowground collections in the open-top chamber (OTC) experiment. The experiment comprises two phases. **Phase 1** (Baseline) took place throughout 2019, during which all preparatory procedures were conducted, including the installation of OTCs and initial collections to establish a comprehensive baseline characterization. Soil and surface ingrowth cores were reset in November 2019, just before the onset of elevated CO_2 conditions. **Phase 2** (Elevated CO_2 experiment) began with CO_2 enrichment. Fine root dynamics in the litter layer were assessed after eight months of elevated CO_2 (July 2020). Fine root dynamics in the soil were evaluated after 12 months of elevated CO_2 (November 2020). At the same time, soil samples were collected to assess root biomass stocks, nutrient concentrations, and microbial activity, including microbial CNP biomass and enzyme activity. The litter decomposition experiment was established in September 2020, with a single collection conducted one year later (September 2021).

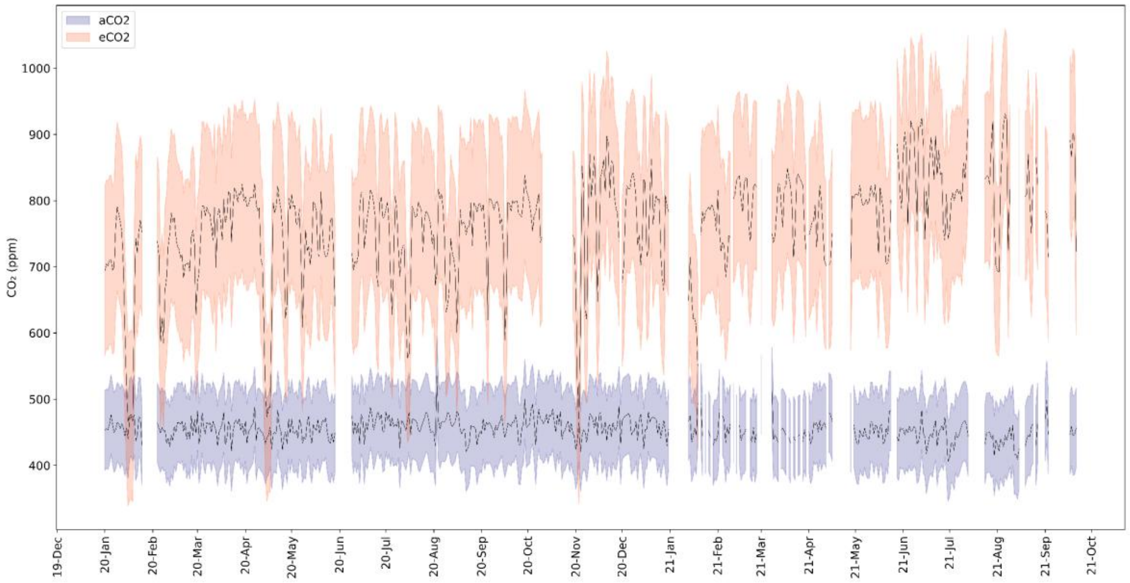

Figure S4 | Concentration of CO_2 inside the Open-top chambers. The lines show the daily average and standard error (n=4), the blue lines represent the ambient CO_2 concentration (aCO_2), and the red line is the elevated CO_2 (eCO_2). The data represent the period between January 2020 and October 2021, where 92% of the time has maintained an increase in CO_2 concentration on average by 309 ppm.

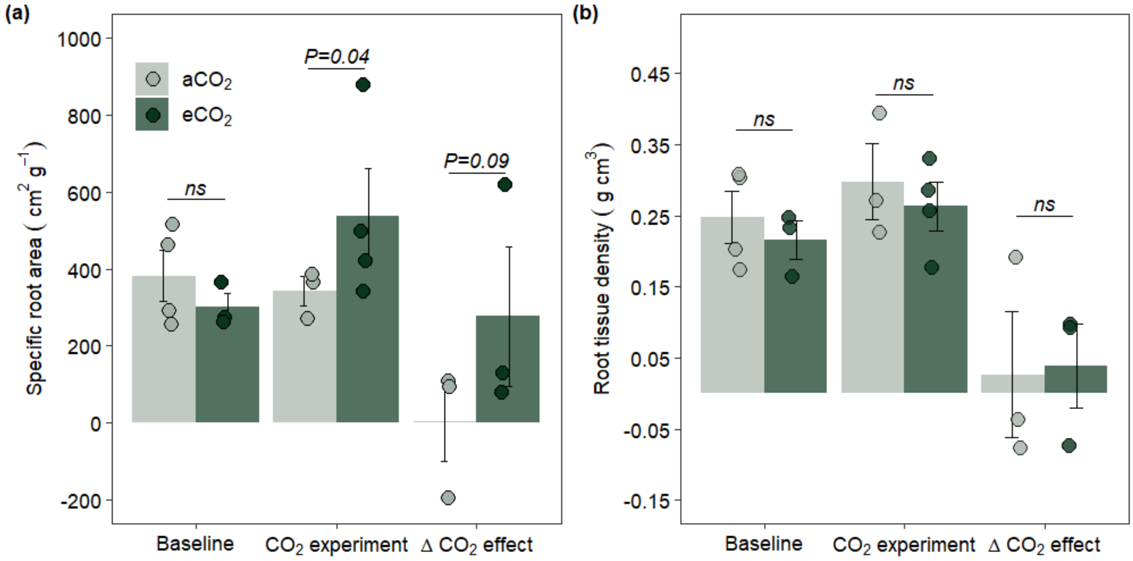

Figure S5 | Effect of Elevated CO_2 on fine root morphological parameters in the litter layer. (a) specific root area (SRA), and (b) root tissue density (RTD). Data represent baseline collections conducted in August 2019, before the start of elevated CO_2 exposure, and subsequent collections following eight months (CO_2 experiment - July 2020). The delta CO_2 (ΔCO_2) effect was quantified as the difference between the experimental CO_2 treatment and its respective baseline (i.e., ambient CO_2 treatment - ambient CO_2 baseline; elevated CO_2 treatment - elevated CO_2 baseline). Statistical analyses were performed using generalized linear mixed models, with distinct models applied to each collection period and variable. Treatment effects were included as fixed factors, while spatial variability was accounted for by incorporating paired OTC as a random factor. The numerical values atop each bar denote the probability of no significant difference between group means. Bars represent mean values ± standard error (n = 4), with individual data points indicating sample distribution within each group.

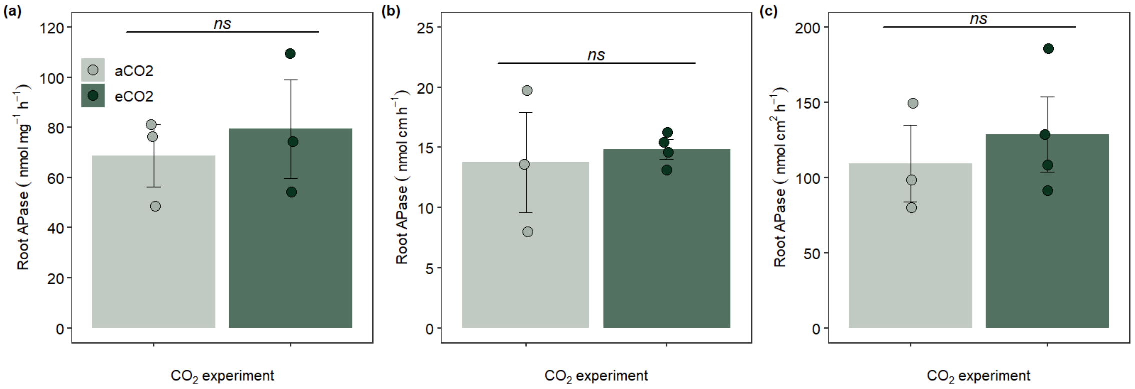

Figure S6| Elevated CO_2 effect on fine root acid phosphatase (APase) activity in the litter layer. Effects of eCO_2 on (a) root APase activity by mg of the dry root, (b) root APase activity by specific root length, and (c) root APase activity by specific root area. Data represents only the collection after eight months under elevated CO_2 conditions (CO_2 experiment - July 2020). Statistical analyses were performed using generalized linear mixed models, with distinct models applied to each collection period and variable. Treatment effects were included as fixed factors, while spatial variability was accounted for by incorporating paired OTC as a random factor. The numerical values atop each bar denote the probability of no significant difference between group means. Bars represent mean values ± standard error (n = 4), with individual data points indicating sample distribution within each group.

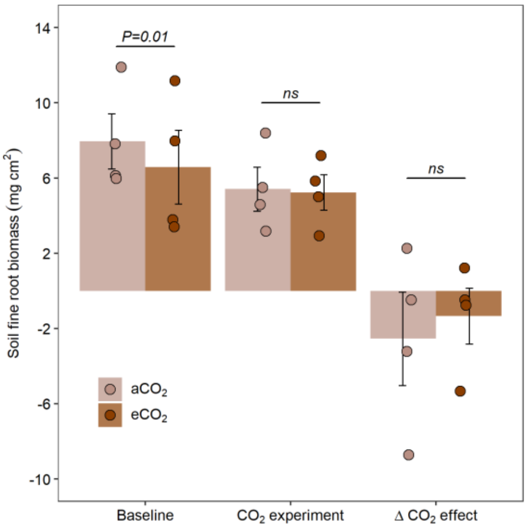

Figure S7 | Effect of Elevated CO_2 on soil fine root biomass. Data represent baseline collections conducted in August 2019, before the start of elevated CO_2 exposure, and subsequent collections following 12 months (CO_2 experiment - November 2020) under elevated CO_2 conditions. The delta CO_2 (ΔCO_2) effect was quantified as the difference between the experimental CO_2 treatment and its respective baseline (i.e., ambient CO_2 treatment - ambient CO_2 baseline; elevated CO_2 treatment - elevated CO_2 baseline). Statistical analyses were performed using generalized linear mixed models, with distinct models applied to each collection period and variable. Treatment effects were included as fixed factors, while spatial variability was accounted for by incorporating paired OTC as a random factor. The numerical values atop each bar denote the probability of no significant difference between group means. Bars represent mean values ± standard error (n = 4), with individual data points indicating sample distribution within each group.

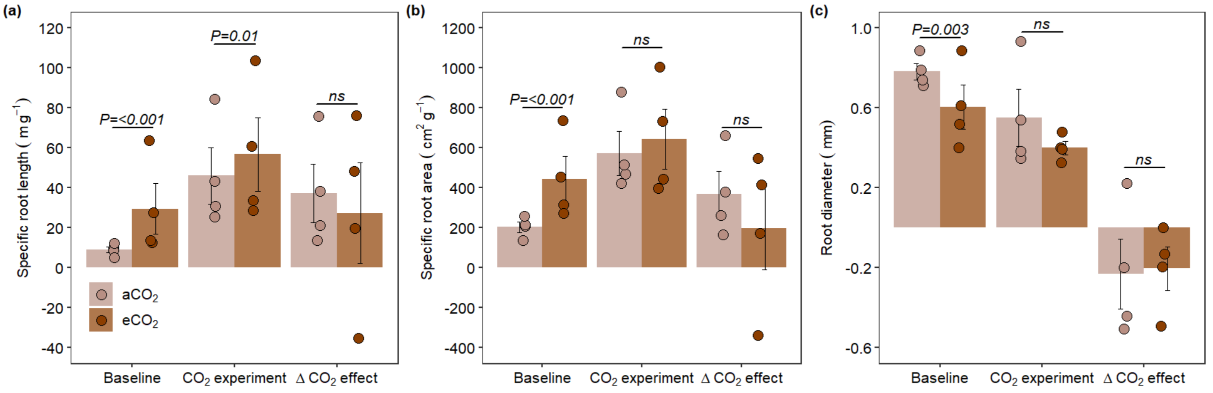

Figure S8 | Effect of elevated CO_2 on fine root morphological parameters in the soil. (a) specific root length (SRL), (b) specific root area (SRA), and (c) root diameter. Data represent baseline collections conducted in August 2019, before the start of elevated CO_2 exposure, and subsequent collections following 12 months (CO_2 experiment - November 2020) under elevated CO_2 conditions. The delta CO_2 (ΔCO_2) effect was quantified as the difference between the experimental CO_2 treatment and its respective baseline (i.e., ambient CO_2 treatment - ambient CO_2 baseline; elevated CO_2 treatment - elevated CO_2 baseline). Statistical analyses were performed using generalized linear mixed models, with distinct models applied to each collection period and variable. Treatment effects were included as fixed factors, while spatial variability was accounted for by incorporating paired OTC as a random factor. The numerical values atop each bar denote the probability of no significant difference between group means. Bars represent mean values ± standard error (n = 4), with individual data points indicating sample distribution within each group.

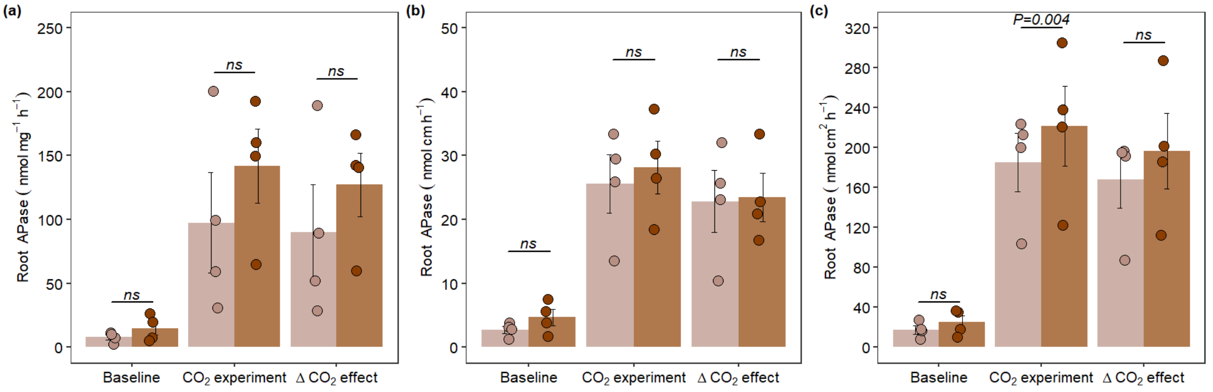

Figure S9 | Elevated CO_2 effect on fine root acid phosphatase (APase) activity in the soil. Effects of eCO_2 on (a) root APase activity by mg of the dry root, (b) root APase activity by specific root length, and (c) root APase activity by specific root area. Data represent baseline collections conducted in August 2019, before the start of elevated CO_2 exposure, and subsequent collections following 12 months (CO_2 experiment - November 2020) under elevated CO_2 conditions. The delta CO_2 (ΔCO_2) effect was quantified as the difference between the experimental CO_2 treatment and its respective baseline (i.e., ambient CO_2 treatment - ambient CO_2 baseline; elevated CO_2 treatment - elevated CO_2 baseline). Statistical analyses were performed using generalized linear mixed models, with distinct models applied to each collection period and variable. Treatment effects were included as fixed factors, while spatial variability was accounted for by incorporating paired OTC as a random factor. The numerical values atop each bar denote the probability of no significant difference between group means. Bars represent mean values ± standard error (n = 4), with individual data points indicating sample distribution within each group.

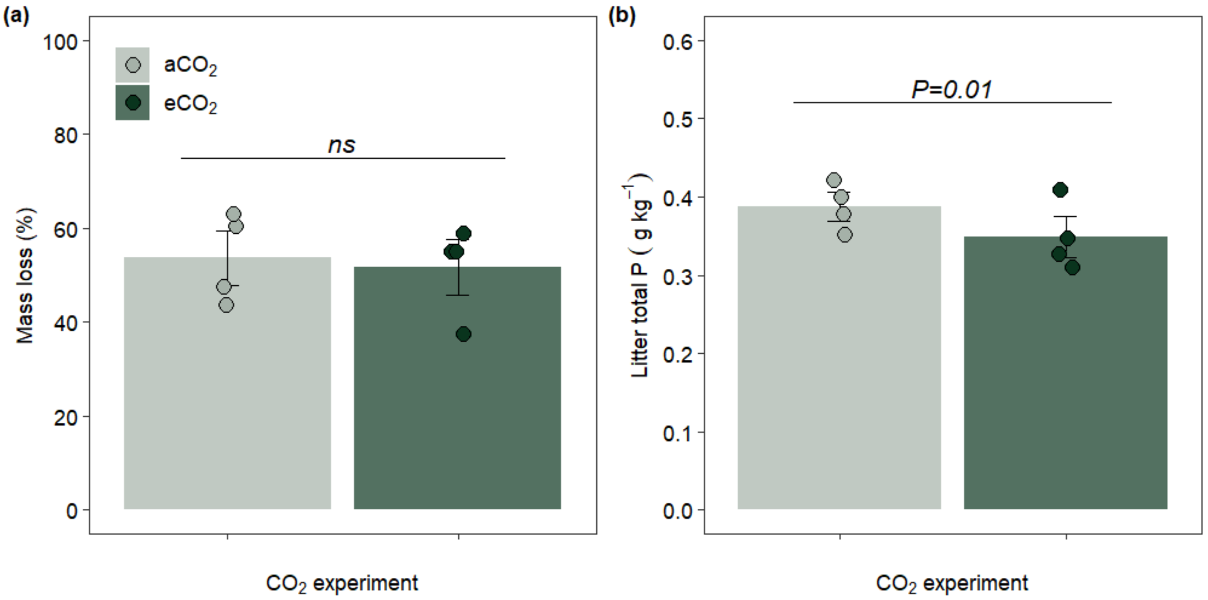

Figure S10 | Effect of elevated CO_2 on leaf litter decomposition and leaf litter P concentration. (a) Leaf litter decomposition represented by mass loss and (b) leaf litter P concentration after one year in the field exposed to elevated CO_2. Statistical analyses were performed using generalized linear mixed models, with distinct models applied to each collection period and variable. Treatment effects were included as fixed factors, while spatial variability was accounted for by incorporating paired OTC as a random factor. The numerical values atop each bar denote the probability of no significant difference between group means. Bars represent mean values ± standard error (n = 4), with individual data points indicating sample distribution within each group.

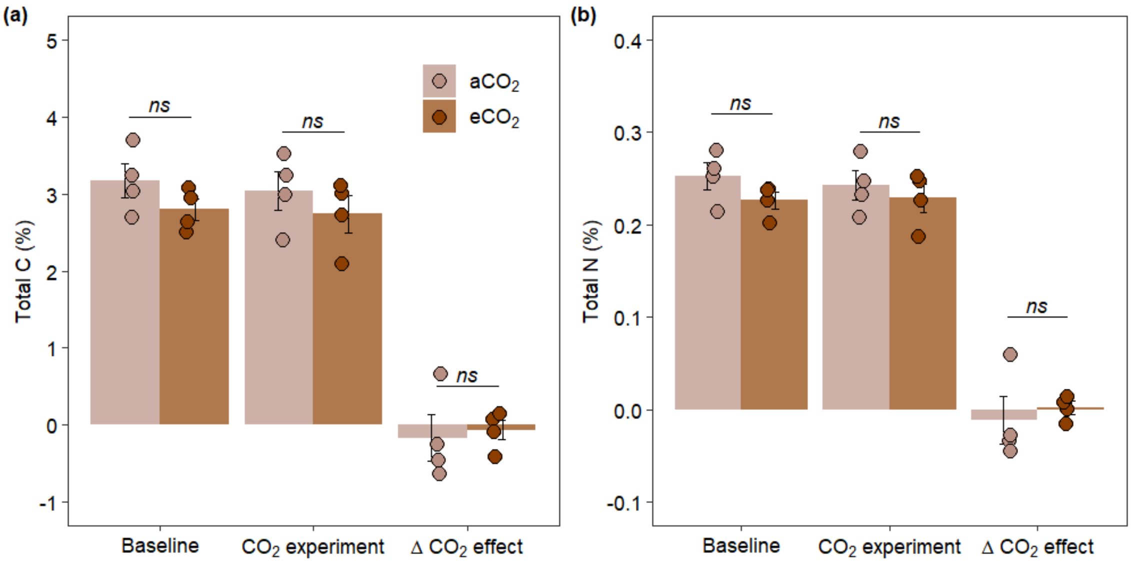

Figure S11 | Effect of elevated CO_2 on soil carbon and nitrogen dynamics. The (a) percent of total soil carbon and (b) the percent of total soil N. Data represent baseline collections conducted in August 2019, before the start of elevated CO_2 exposure, and subsequent collections following 12 months (CO_2 experiment - November 2020) under elevated CO_2 conditions. The delta CO_2 (ΔCO_2) effect was quantified as the difference between the experimental CO_2 treatment and its respective baseline (i.e., ambient CO_2 treatment - ambient CO_2 baseline; elevated CO_2 treatment - elevated CO_2 baseline). Statistical analyses were performed using generalized linear mixed models, with distinct models applied to each collection period and variable. Treatment effects were included as fixed factors, while spatial variability was accounted for by incorporating paired OTC as a random factor. The numerical values atop each bar denote the probability of no significant difference between group means. Bars

represent mean values ± standard error (n = 4), with individual data points indicating sample distribution within each group.

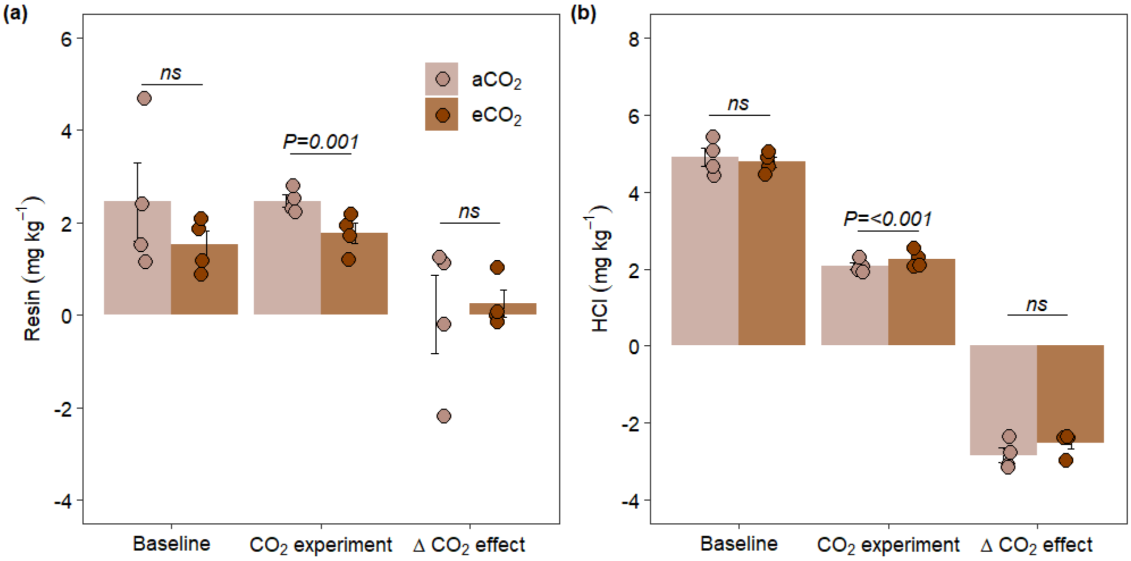

Figure S12 | Elevated CO_2 effect on soil phosphorus fractions. The soil P fractions were obtained using the Hedley soil P fractionation methodology (see "Methods" for details). The resin P fraction (a) was the first fraction obtained by a membrane of anion exchange, followed by the extraction of (b) the hydrogen chloride fraction (HCl). Data represent baseline collections conducted in February 2019, before the start of elevated CO_2 exposure, and subsequent collections following 12 months (CO_2 experiment - November 2020) under elevated CO_2 conditions. The delta CO_2 (ΔCO_2) effect was quantified as the difference between the experimental CO_2 treatment and its respective baseline (i.e., ambient CO_2 treatment - ambient CO_2 baseline; elevated CO_2 treatment - elevated CO_2 baseline). Statistical analyses were performed using generalized linear mixed models, with distinct models applied to each collection period and variable. Treatment effects were included as fixed factors, while spatial variability was accounted for by incorporating paired OTC as a random factor. The numerical values atop each bar denote the probability of no significant difference between group means. Bars represent mean values ± standard error (n = 4), with individual data points indicating sample distribution within each group.

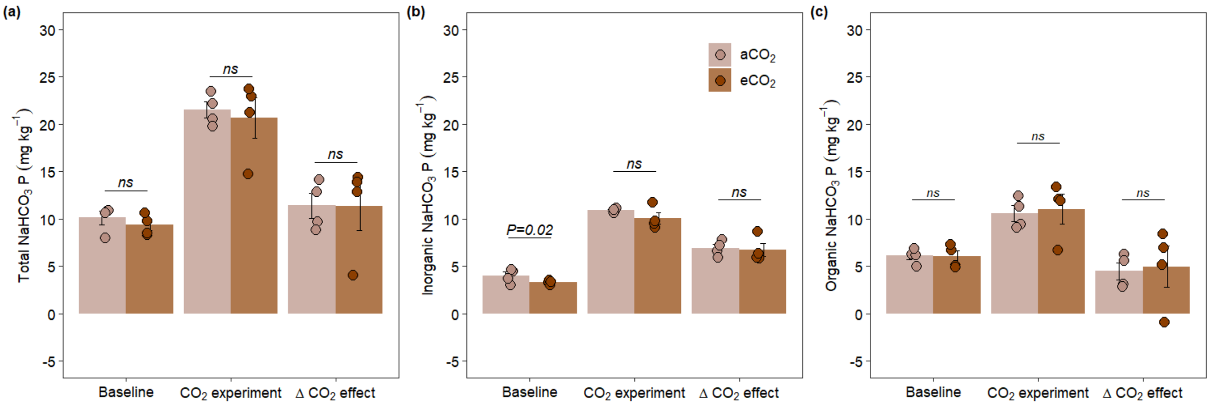

Figure S13 | Elevated CO_2 effect on soil phosphorus fractions. The soil P fractions were obtained using the Hedley P fractionation methodology (see methods section for details). The $NaHCO_3$ represents the fractions of P resulting from bicarbonate extraction: (a) total bicarbonate P, (b) inorganic bicarbonate P, and (c) organic bicarbonate P. Data represent baseline collections conducted in February 2019, before the start of elevated CO_2 exposure, and subsequent collections following 12 months (CO_2 experiment - November 2020) under elevated CO_2 conditions. The delta CO_2 (ΔCO_2) effect was quantified as the difference between the experimental CO_2 treatment and its respective baseline (i.e., ambient CO_2 treatment - ambient CO_2 baseline; elevated CO_2 treatment - elevated CO_2 baseline). Statistical analyses were performed using generalized linear mixed models, with distinct models applied to each collection period and variable. Treatment effects were included as fixed factors, while

spatial variability was accounted for by incorporating paired OTC as a random factor. The numerical values atop each bar denote the probability of no significant difference between group means. Bars represent mean values ± standard error (n = 4), with individual data points indicating sample distribution within each group.

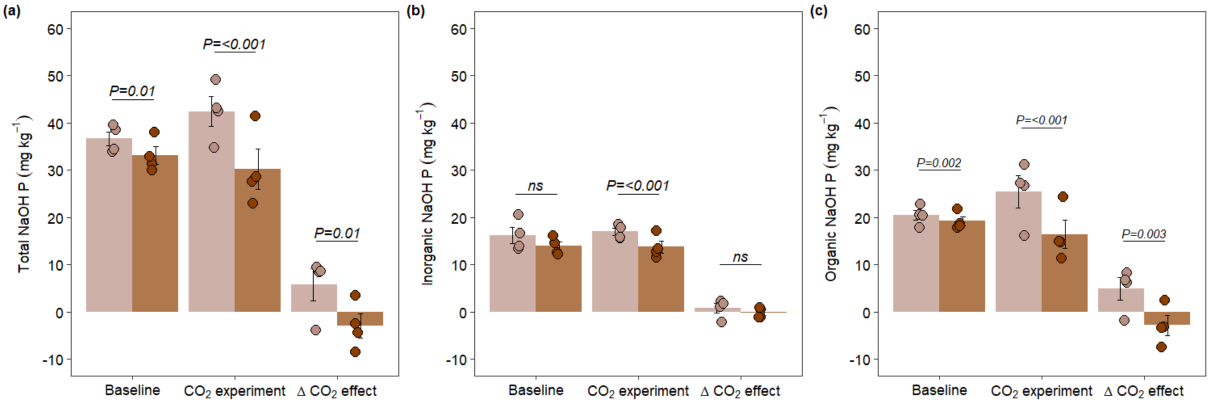

Figure S14 | Elevated CO_2 effect on soil phosphorus fractions. The soil P fractions were obtained using the Hedley soil P fractionation methodology (see "Methods" for details). The NaOH P fractions resulted from the hydroxide extraction: (a) total hydroxide P, (b) inorganic hydroxide P, and (c) organic hydroxide P. Data represent baseline collections conducted in February 2019, before the start of elevated CO_2 exposure, and subsequent collections following 12 months (CO_2 experiment - November 2020) under elevated CO_2 conditions. The delta CO_2 (ΔCO_2) effect was quantified as the difference between the experimental CO_2 treatment and its respective baseline (i.e., ambient CO_2 treatment - ambient CO_2 baseline; elevated CO_2 treatment - elevated CO_2 baseline). Statistical analyses were performed using generalized linear mixed models, with distinct models applied to each collection period and variable. Treatment effects were included as fixed factors, while spatial variability was accounted for by incorporating OTC as a random factor. The numerical values atop each bar denote the probability of no significant difference between group means. Bars represent mean values ± standard error (n = 4), with individual data points indicating sample distribution within each group.

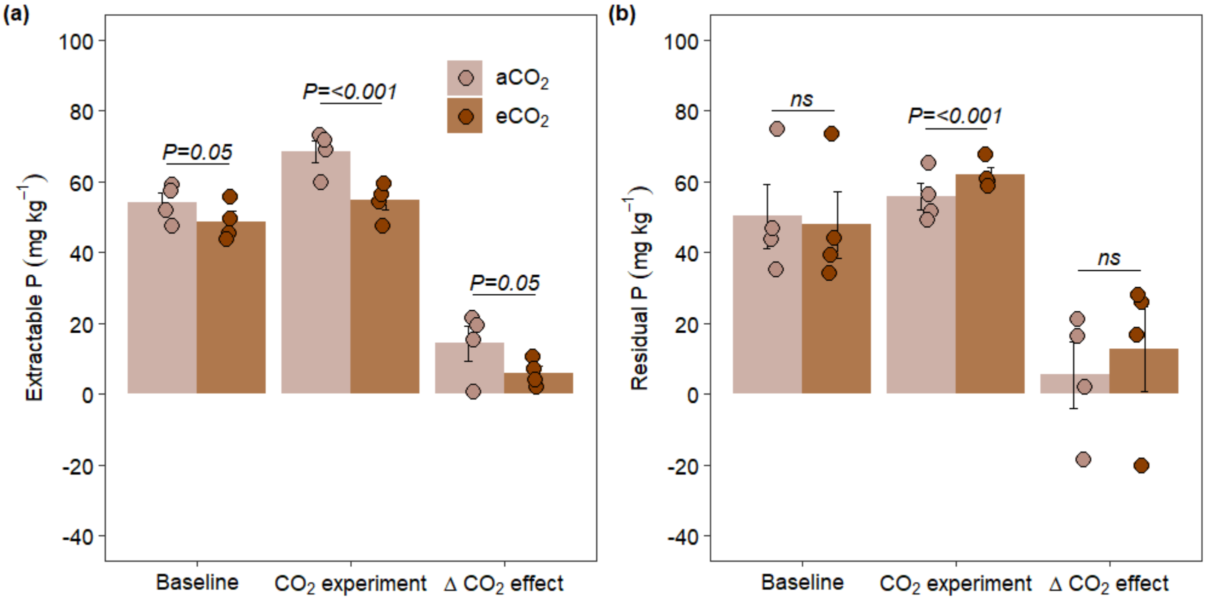

Figure S15 | Elevated CO_2 effect on soil phosphorus fractions. The soil P fractions were obtained using the Hedley soil P fractionation methodology (see "Methods" for details). The (a) extractable P represents the total P, which may be available and represented by the sum of the organic and inorganic fractions ($NaHCO_3$ and NaOH) plus the resin and HCl. The (b) residual P was obtained by the difference between total and extractable P and represents the unavailable P. Data represent baseline collections conducted in February 2019, before the start of elevated CO_2 exposure, and subsequent collections following 12 months (CO_2 experiment - November 2020) under elevated CO_2 conditions. The delta CO_2 (ΔCO_2) effect was quantified as the difference between the experimental

CO_2 treatment and its respective baseline (i.e., ambient CO_2 treatment - ambient CO_2 baseline; elevated CO_2 treatment - elevated CO_2 baseline; see Methods). Statistical analyses were performed using generalized linear mixed models, with distinct models applied to each collection period and variable. Treatment effects were included as fixed factors, while spatial variability was accounted for by incorporating paired OTC as a random factor. The numerical values atop each bar denote the probability of no significant difference between group means. Bars represent mean values ± standard error (n = 4), with individual data points indicating sample distribution within each group.

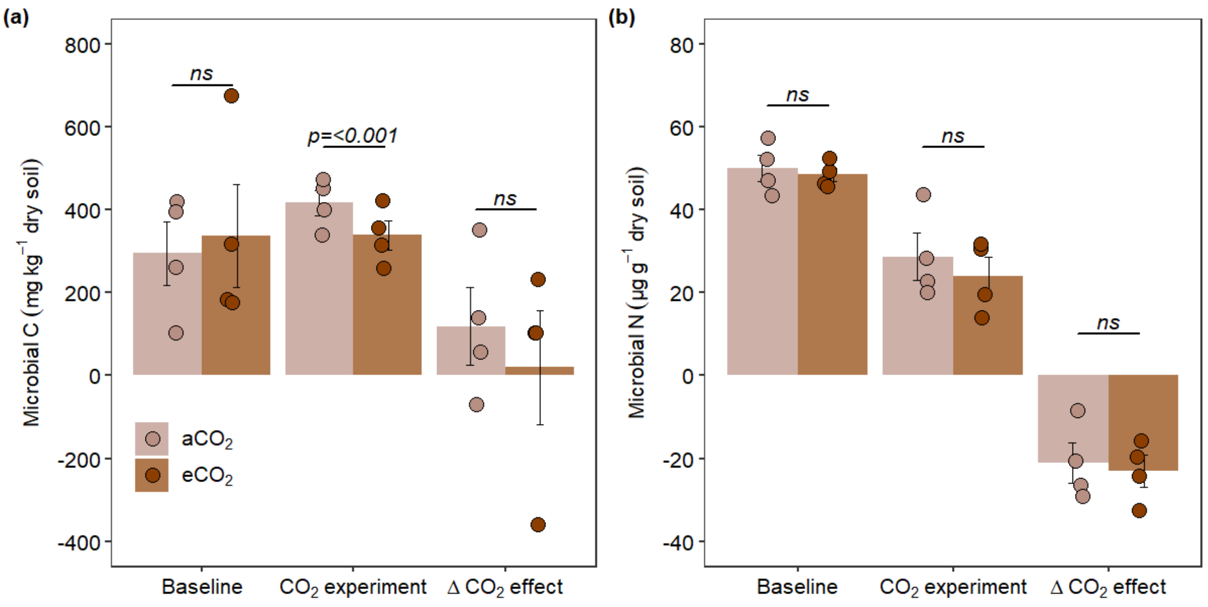

Figure S16 | Elevated CO_2 effect on soil microbial biomass. The total amount of (a) carbon and (b) nitrogen, is immobilized on microbial biomass. Data represent baseline collections conducted in August 2019, before the start of elevated CO_2 exposure, and subsequent collections following 12 months (CO_2 experiment - November 2020) under elevated CO_2 conditions. The delta CO_2 (ΔCO_2) effect was quantified as the difference between the experimental CO_2 treatment and its respective baseline (i.e., ambient CO_2 treatment - ambient CO_2 baseline; elevated CO_2 treatment - elevated CO_2 baseline). Statistical analyses were performed using generalized linear mixed models, with distinct models applied to each collection period and variable. Treatment effects were included as fixed factors, while spatial variability was accounted for by incorporating paired OTC as a random factor. The numerical values atop each bar denote the probability of no significant difference between group means. Bars represent mean values ± standard error (n = 4), with individual data points indicating sample distribution within each group.

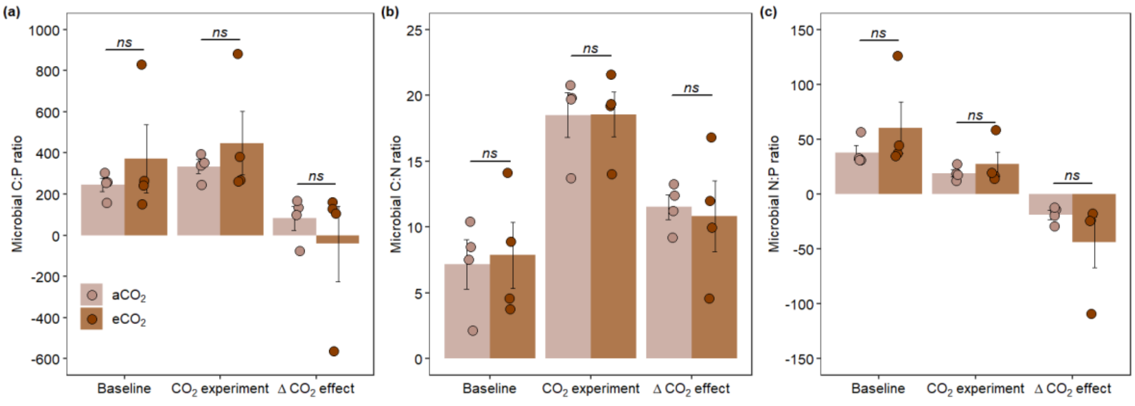

Figure S17 | Elevated CO_2 effect on the stoichiometry of soil microbial biomass. The ratio of (a) carbon and phosphorus, (b) carbon and nitrogen, and (c) nitrogen and phosphorus, respectively, in microbial biomass. Data represent baseline collections conducted in August 2019, before the start of elevated CO_2 exposure, and subsequent collections following 12 months (CO_2 experiment - November 2020) under elevated CO_2 conditions. The delta

CO_2 (ΔCO_2) effect was quantified as the difference between the experimental CO_2 treatment and its respective baseline (i.e., ambient CO_2 treatment - ambient CO_2 baseline; elevated CO_2 treatment - elevated CO_2 baseline). Statistical analyses were performed using generalized linear mixed models, with distinct models applied to each collection period and variable. Treatment effects were included as fixed factors, while spatial variability was accounted for by incorporating paired OTC as a random factor. The numerical values atop each bar denote the probability of no significant difference between group means. Bars represent mean values ± standard error (n = 4), with individual data points indicating sample distribution within each group.

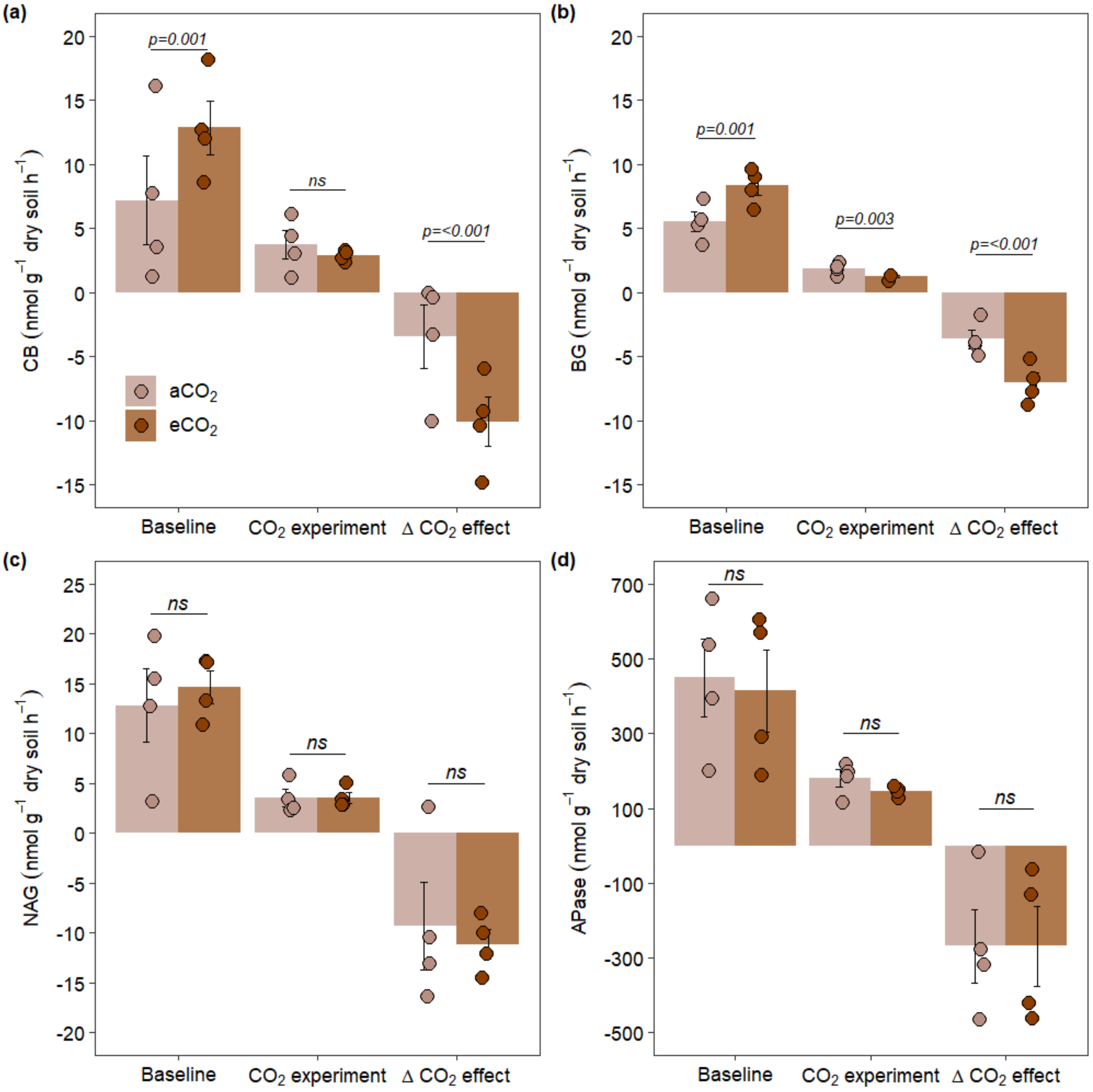

Figure S18 | Elevated CO_2 effect on soil extracellular enzyme activity. The enzymes (a) cellobiosidase (CB) and (b) β-glucosidase (BG) are responsible for hydrolyzing the carbon molecules, (c) β-1,4-N-acetylglucosaminidase (NAG) is responsible for the hydrolysis of nitrogen, and (b) acid phosphatase (APase) is responsible for the hydrolysis of organic phosphorus. Data represent baseline collections conducted in August 2019, before the start of elevated CO_2 exposure, and subsequent collections following 12 months (CO_2 experiment - November 2020) under elevated CO_2 conditions. The delta CO_2 (ΔCO_2) effect was quantified as the difference between the experimental CO_2 treatment and its respective baseline (i.e., ambient CO_2 treatment - ambient CO_2 baseline; elevated CO_2 treatment - elevated CO_2 baseline). Statistical analyses were performed using generalized linear mixed models, with distinct models applied to each collection period and variable. Treatment effects were included as fixed factors, while spatial variability was accounted for by incorporating paired OTC as a random factor. The numerical values atop each bar denote the probability of no significant difference between group

means. Bars represent mean values ± standard error (n = 4), with individual data points indicating sample distribution within each group.

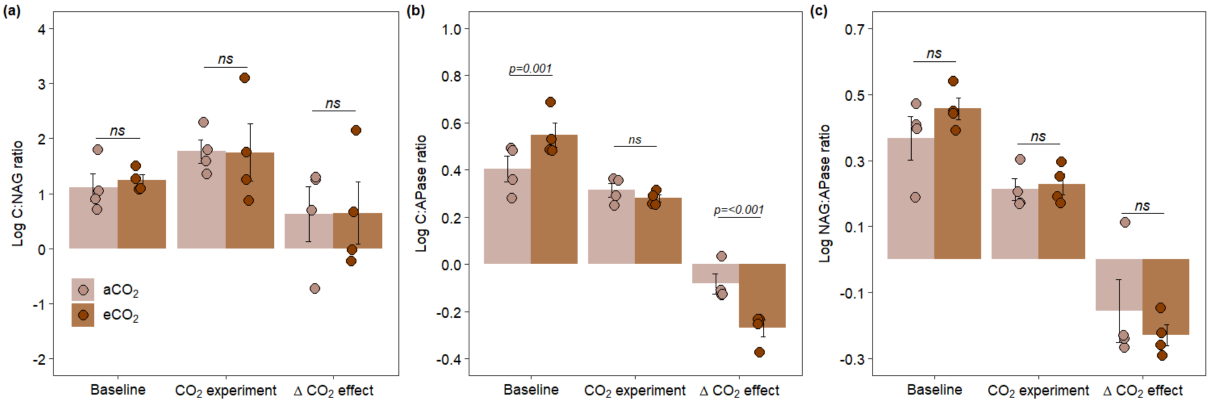

Figure S19 | Elevated CO_2 effect on soil extracellular enzymes stoichiometry. The stoichiometry was obtained using the log ratio. The carbon enzymes are represented by the sum of cellobiosidase (CB) and β-1,4-glucosidase (BG) activity. The ratio of investment for (a) carbon and nitrogen acquisition investments is represented by the ratio of the total of C enzymes and β-1,4-N-acetylglucosaminidase (NAG), the log C: APase ratio (b) represents the total of carbon enzymes by soil acid phosphatase and means the ratio of microbial carbon investment on carbon and phosphorus acquisition, and (c) the nitrogen and phosphorus acquisition investments are represented by the ratio of β-1,4-N-acetylglucosaminidase (NAG) and acid phosphatase (APase). Data represent baseline collections conducted in August 2019, before the start of elevated CO_2 exposure, and subsequent collections following 12 months (CO_2 experiment - November 2020) under elevated CO_2 conditions. The delta CO_2 (ΔCO_2) effect was quantified as the difference between the experimental CO_2 treatment and its respective baseline (i.e., ambient CO_2 treatment - ambient CO_2 baseline; elevated CO_2 treatment - elevated CO_2 baseline). Statistical analyses were performed using generalized linear mixed models, with distinct models applied to each collection period and variable. Treatment effects were included as fixed factors, while spatial variability was accounted for by incorporating OTC as a random factor. The numerical values atop each bar denote the probability of no significant difference between group means. Bars represent mean values ± standard error (n = 4), with individual data points indicating sample distribution within each group.

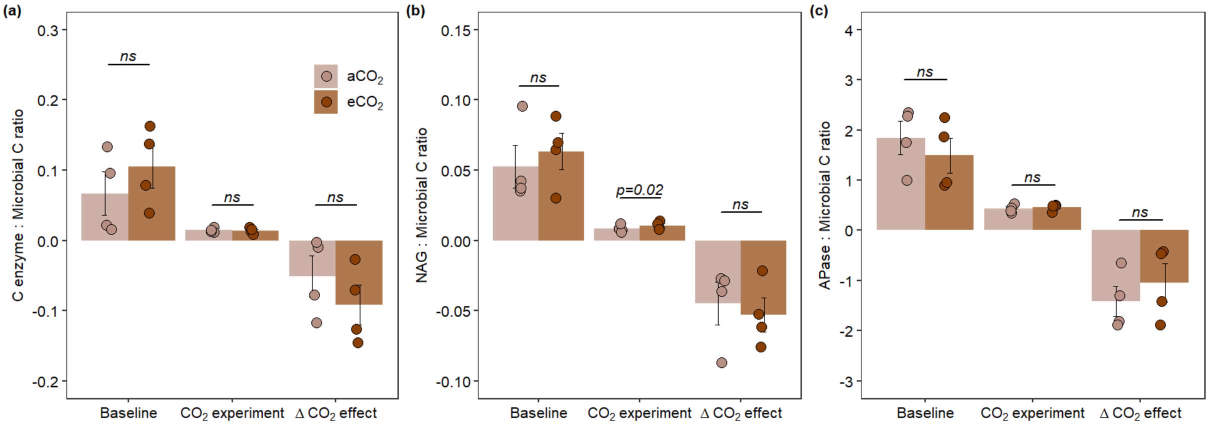

Figure S20| Elevated CO_2 effect on the stoichiometry of soil enzymes and C microbial biomass. The carbon enzymes are represented by the sum of cellobiosidase (CB) and β-1,4-glucosidase (BG) activity. (a) The ratio of carbon enzymes and carbon microbial biomass; (b) the ratio of nitrogen enzyme activity (β-1,4-N-acetylglucosaminidase) and carbon microbial biomass; (c) the ratio of phosphorus enzyme activity (acid phosphatase) and carbon microbial biomass. Data represent baseline collections conducted in August 2019, before the start of elevated CO_2 exposure, and subsequent collections following 12 months (CO_2 experiment - November 2020) under elevated CO_2 conditions. The delta CO_2 (ΔCO_2) effect was quantified as the difference between the experimental CO_2 treatment and its respective baseline (i.e., ambient CO_2 treatment - ambient CO_2 baseline; elevated CO_2 treatment - elevated CO_2 baseline). Statistical analyses were performed using generalized linear mixed models, with distinct models applied to each collection period and variable. Treatment effects were included

as fixed factors, while spatial variability was accounted for by incorporating paired OTC as a random factor. The numerical values atop each bar denote the probability of no significant difference between group means. Bars represent mean values ± standard error ($n = 4$), with individual data points indicating sample distribution within each group.
