# Supplementary material for: Amazonian understory forests change phosphorus acquisition strategies under elevated CO2
Source: Nat Commun. 2026 Apr 28;17:3740. doi: 10.1038/s41467-026-72098-0 (PMC13125542; doi:10.1038/s41467-026-72098-0)
Supplement: Supplementary file 3 — Reporting Summary [file 41467_2026_72098_MOESM3_ESM.pdf]

nature portfolio

Corresponding author(s): Nathielly P. Martins and Lucia Fuchslueger

Last updated by author(s): Mar 24, 2026

Reporting Summary

Nature Portfolio wishes to improve the reproducibility of the work that we publish. This form provides structure for consistency and transparency in reporting. For further information on Nature Portfolio policies, see our Editorial Policies and the Editorial Policy Checklist.

Statistics

For all statistical analyses, confirm that the following items are present in the figure legend, table legend, main text, or Methods section.

n/a	Confirmed	
☐	☒	The exact sample size (*n*) for each experimental group/condition, given as a discrete number and unit of measurement
☐	☒	A statement on whether measurements were taken from distinct samples or whether the same sample was measured repeatedly
☐	☒	The statistical test(s) used AND whether they are one- or two-sided<br>*Only common tests should be described solely by name; describe more complex techniques in the Methods section.*
☐	☒	A description of all covariates tested
☐	☒	A description of any assumptions or corrections, such as tests of normality and adjustment for multiple comparisons
☐	☒	A full description of the statistical parameters including central tendency (e.g. means) or other basic estimates (e.g. regression coefficient) AND variation (e.g. standard deviation) or associated estimates of uncertainty (e.g. confidence intervals)
☐	☒	For null hypothesis testing, the test statistic (e.g. *F*, *t*, *r*) with confidence intervals, effect sizes, degrees of freedom and *P* value noted<br>*Give P values as exact values whenever suitable.*
☒	☐	For Bayesian analysis, information on the choice of priors and Markov chain Monte Carlo settings
☒	☐	For hierarchical and complex designs, identification of the appropriate level for tests and full reporting of outcomes
☐	☒	Estimates of effect sizes (e.g. Cohen's *d*, Pearson's *r*), indicating how they were calculated

Our web collection on statistics for biologists contains articles on many of the points above.

Software and code

Policy information about availability of computer code

Data collection	Not applicable
Data analysis	All data analyses were performed using the software R, version 4.5.2 from 2025.

For manuscripts utilizing custom algorithms or software that are central to the research but not yet described in published literature, software must be made available to editors and reviewers. We strongly encourage code deposition in a community repository (e.g. GitHub). See the Nature Portfolio guidelines for submitting code & software for further information.

Data

Policy information about availability of data

All manuscripts must include a data availability statement. This statement should provide the following information, where applicable:

- Accession codes, unique identifiers, or web links for publicly available datasets
- A description of any restrictions on data availability
- For clinical datasets or third party data, please ensure that the statement adheres to our policy

The datasets generated and analyzed in this study will be available upon publication at Zenodo repository at DOI:10.5281/zenodo.18495501

April 2023

Research involving human participants, their data, or biological material

Policy information about studies with human participants or human data. See also policy information about sex, gender (identity/presentation), and sexual orientation and race, ethnicity and racism.

Reporting on sex and gender	not applicable
Reporting on race, ethnicity, or other socially relevant groupings	not applicable
Population characteristics	not applicable
Recruitment	not applicable
Ethics oversight	not applicable

Note that full information on the approval of the study protocol must also be provided in the manuscript.

Field-specific reporting

Please select the one below that is the best fit for your research. If you are not sure, read the appropriate sections before making your selection.

☐ Life sciences ☐ Behavioural & social sciences ☒ Ecological, evolutionary & environmental sciences

For a reference copy of the document with all sections, see nature.com/documents/nr-reporting-summary-flat.pdf

Ecological, evolutionary & environmental sciences study design

All studies must disclose on these points even when the disclosure is negative.

Study description	This study investigates how understory plant communities in phosphorus-poor Amazonian forests adapt their nutrient acquisition strategies in response to elevated atmospheric CO_2 (eCO_2). Using in situ open-top chamber (OTC) experiments that increased CO_2 concentrations by ~300 ppm in four OTC and used others four as a control with the CO2 concentration ambient, we examined plant and microbial responses in both the soil and litter layers.
Research sample	Two soil and root samples were collected from each OTC in both the litter layer and the soil (0-15 cm deep) during the baseline period and after the onset of elevated CO_2 treatment. For more detailed information on sample collection procedures, please refer to the Methods section
Sampling strategy	To account for spatial variability within each OTC, the two samples collected per chamber were averaged. Based on the experimental design, the sample size for all measurements is n = 4. Detailed sampling procedures for the various root and soil variables are provided in the Methods section.
Data collection	Data collection was led primarily by Nathielly P. Martins, with additional contributions from several co-authors as detailed in the "author contribution statement".
Timing and spatial scale	Data collection started in 2019 with a comprehensive baseline assessment across all open-top chambers (OTCs). The elevated CO_2 treatment stat in November 2019, and data reflecting this treatment were collected throughout 2020 and 2021. For a detailed timeline of measurements for each variable, refer to Figure S3 in the Supplementary Material.
Data exclusions	Data were excluded when incorrect laboratory procedures were identified, primarily affecting the microbial biomass and extracellular enzyme datasets
Reproducibility	All data necessary to replicate the experiment are reproducible and will be made available.
Randomization	OTC pairs were included as a random factor to account for spatial variability
Blinding	not applicable

Did the study involve field work? ☒ Yes ☐ No

Field work, collection and transport

Field conditions	The study was conducted on a plateau, and using the Open Top Chamber (OTC) was possible to consider the effecf of elevated CO2 in the understory trees. The soil is a highly weathered, clay-rich Geric Ferralsol (67.7% clay, 19.9% sand, 12.3% silt), characterized by

	low total phosphorus and rock-derived nutrient concentrations, and an acidic pH of 3.94. The region receives an average annual rainfall of approximately 2,500 mm, with the wet season occurring from December to May and a pronounced dry season from August to September (typically <100 mm of rainfall). Mean annual temperatures range between 24°C and 27°C."
Location	The open-top chamber (OTC) experiment was conducted within the AmazonFACE experimental area (−2°35′40.29″, −60°12′28.69″), located in the Cuieiras Experimental Reserve, approximately 70 km north of Manaus, in the Central Amazon (Amazonas, Brazil).
Access & import/export	Not applicable, the experiment is located on a reasearch experimental station.
Disturbance	The study site is located in undisturbed, old-growth Amazonian terra firme forest. No recent anthropogenic or natural disturbances were recorded prior to or during the experiment.

Reporting for specific materials, systems and methods

We require information from authors about some types of materials, experimental systems and methods used in many studies. Here, indicate whether each material, system or method listed is relevant to your study. If you are not sure if a list item applies to your research, read the appropriate section before selecting a response.

Materials & experimental systems

n/a	Involved in the study
☒	☐ Antibodies
☒	☐ Eukaryotic cell lines
☒	☐ Palaeontology and archaeology
☒	☐ Animals and other organisms
☒	☐ Clinical data
☒	☐ Dual use research of concern
☒	☐ Plants

Methods

n/a	Involved in the study
☒	☐ ChIP-seq
☒	☐ Flow cytometry
☒	☐ MRI-based neuroimaging

Plants

Seed stocks	not applicable
Novel plant genotypes	not applicable
Authentication	not applicable
